# The Pathogenesis of Autism

**DOI:** 10.4137/cpath.s1143

**Published:** 2008-09-18

**Authors:** Timothy John Watts

**Affiliations:** Barts and the London School of Medicine and Dentistry, Turner Street, London, E1 2AD, U.K

**Keywords:** autism, neural migration, dendritic, neuroimmune, mirror neurone

## Abstract

Autism is well known as a complex developmental disorder with a seemingly confusing and uncertain pathogenesis. The definitive mechanisms that promote autism are poorly understood and mostly unknown, yet available theories do appear to focus on the disruption of normal cerebral development and its subsequent implications on the functional brain unit. This mini-review aims solely to discuss and evaluate the most prominent current theories regarding the pathogenesis of autism. The main conclusion is that although there is not a clear pathway of mechanisms directed towards a simple pathogenesis and an established link to autism on the symptomatic level; there are however several important theories (neural connectivity, neural migration, excitatory-inhibitory neural activity, dendritic morphology, neuroimmune; calcium signalling and mirror neurone) which appear to offer an explanation to how autism develops. It seems probable that autism’s neurodevelopmental defect is ‘multi-domain’ in origin (rather than a single anomaly) and is hence distributed across numerous levels of study (genetic, immunopathogenic, etc.). A more definitive understanding of the pathogenesis could facilitate the development of better treatments for this complex psychiatric disorder.

## Introduction

Autism is one of the pervasive developmental disorders (or autism spectrum disorders) and a commonly diagnosed condition in child and adolescent psychiatry, which was initially described by the psychiatrist Leo Kanner in 1943 (Kanner, 1943). Childhood autism is broadly defined by the presence of abnormal and impaired development, which manifests into a series of clinically relevant areas (or symptoms). The current prevalence of autism spectrum disorders is estimated at 6 per 1000 (1.3 per 1000 for autism itself), with a male/female ratio of 3–4:1 (Fombonne, 2005). Typically the onset of autism is before 36 months of age (WHO, 2005). Interestingly autism is a highly genetic neuropsychiatric disorder with concordance rates of 82%–92% in monozygotic twins (compared with 1%–10% in dizyogtic twins) (Persico and Bourgeron, 2006). Autism presents with a triad of core symptoms which include: (1) A qualitative impairment of social interaction (an inability to relate to others often with lack of eye contact); (2) Stereotypical, ritualistic, repetitive, restrictive patterns of interests, behaviours and activities and; (3) Major defects in language development and in other communication skills (WHO 2005; Miall et al. 2007). These rather distinctive set of symptoms additionally form the basis of the diagnosis for autism. Affected children also manifest with other non-specific symptoms including: unusual sensory perception skills and experiences, motor clumsiness (and problems with proprioception) and insomnia. The majority of autistic children also have limited intelligence (IQ > 100 in 5%). Strongly considered differentials for the diagnosis include childhood schizophrenia, learning disability and deafness (Fombonne, 2005; WHO, 2005).

The aetiology of autism is complex and is likely to be of multifactorial descent encompassing both genetic predisposition and environmental factors (Trottier et al. 1999). Potential genetic disorders include fragile X syndrome and tuberous sclerosis (Freitag, 2007). The environmental causative element can be effectively separated into pre-natal, peri-natal and post-natal factors. Implicated prenatal factors include congenital rubella syndrome (secondary to rubella infection), teratogen exposure (e.g. thalidomide) and pesticide exposure. Peri-natal factors are associated with obstetric conditions like low birth weight, abnormal gestation length (pre-term delivery) and birth asphyxia (hypoxic-ischaemic insult) (Kolevzon, 2007). Post-natal factors encompass a wide range of insults including autoimmune disease (Ashwood and van de Water, 2004), leaky gut syndrome (Johnson, 2006), viral infection, amygdala developmental failure (Schultz, 2005), oxidative stress (Kern and Jones, 2006), vitamin D deficiency (Cannell, 2007), heavy metal toxicity e.g. mercury (Davidson et al. 2004), and the controversial MMR vaccine (Wakefield et al. 1998). Although the aetiology of autism is a highly contemporary area of research in psychiatry and neuroscience, the focus of this concise review is to consider the pathogenesis of autism, an area which lacks great certainty with regard to the exact underlying neurobiological mechanisms which promote this developmental disorder.

The mechanisms that lead to autism are at best poorly understood, however they do centre around the disruption of normal cerebral development and its subsequent implications on the functional brain unit (although the exact link to the classic triad of core symptoms remains unascertained). Numerous neuropsychiatry papers attribute the pathogenesis of autism specifically to ‘localised’ anomalies (i.e. of neural migration or connectivity), which have the potential to detrimentally effect CNS structure and function (Casanova, 2007; Persico and Bourgeron, 2006). The following aims to summarise and discuss the current debated theories on autism’s pathogenesis with an emphasis on the cellular and molecular neurobiology where relevant.

## Neural Connectivity

One specific theory emphasises that early brain overgrowth and neural overconnectivity are key in the pathogenesis. It is speculated that excess neuron numbers (inducing cerebral overgrowth) may promote defects in neural patterning and wiring, with subsequent over energetic short-distance cortical interactions hindering long-distance interactions that communicate between critical brain regions. Suggesting a more diffuse pathology across a multitude of cortical areas rather than just a single confined defect. This neuroanatomical anomaly has the potential to underlie the deficits in socio-emotional and communicational function observed in autism (Courchesne et al. 2007).

Conversely another related theory postulates that the neuropathological basis of disrupted cognition in autism is linked to reduced intracortical connectivity which subsequently promotes a lower degree of information integration across multiple cortical regions (Just et al. 2007; Minshew and Williams, 2007). Whether or not the pathogenesis is due to over- or underconnectivity it is quite probable that general neural connectivity and inter-neural communication and coordination are fundamental contributors to the mechanism of autism.

## Neural Migration

Cerebral cortical malformations observed in autism may result from defective neural migration to the cerebral cortex during the first 6 months of gestation. The subsequent cortical dysgenesis seen in autism patients with disturbed neural migration includes among others; thickened cortex, high neuronal density, poor grey-white matter boundaries and ectopic grey matter (Schmitz and Rezaie, 2008). This hypothesis is additionally supported by the fact that decreased levels of Reelin (an extracellular matrix protein that contributes in events that are crucial for neuronal migration and cellular positioning) have been observed in post-mortem cerebellar tissue from autistic patients (Magdaleno et al. 2002; Persico and Bourgeron, 2006).

## Excitatory-Inhibitory Neural Activity

The theory of unbalanced excitability-inhibitory networks in autism also carries certain credibility. Available research concludes that chromosomal rearrangements involving GABA receptor gene clusters are heavily implicated, promoting abnormal CNS system excitability and function. The additional role of glutamate receptors in synaptic maintenance also shows potential relevance to autism’s pathophysiology (Dykens et al. 2004; Schmitz and Rezaie, 2008). Yet it remains unascertained to how exactly abnormalities in neuronal excitation and inhibition influence the development of autism on the symptomatic level. In addition, the mechanism suffers itself from the lack of certainty with regard to whether GABA or glutamate receptor dysfunction underlies the observed excitatory abnormalities.

## Dendritic Morphology

The abnormal assembly of synapses and dendritic spines may also be a contributing factor in autism’s pathogenesis. Notably, it has been found that autistic brains feature increased numbers of long, thin, dendritic spines (Minshew and Williams, 2007; Pickett and London, 2005). Crucial to dendritic morphology are the scaffolding proteins which mediate connectivity between membrane proteins and cytoskeleton. Specifically the SHANK3 gene which encodes a synaptic scaffolding protein involved in the induction and maintenance of dendritic spines has been observed deleted in autism patients (Boeckers et al. 2002).

## Neuroimmune Disturbances

The topic of neuroimmune disturbance and its impact on the pathogenesis of autism has also been widely researched. Immune disorders noted in autistic patients include among others: abnormal T-helper cell type 1/2 responses, general suppression of cell-mediated immunity, subnormal levels of CD4+ lymphocytes, imbalance of antibody levels and reduced natural killer cell functions (Cohly and Panja, 2005; Kern and Jones, 2006). Autoimmunity could also be linked to autism following the finding of autoantibodies (i.e. IgG) against nervous system proteins, and additionally an immunogenetic association related to the presence of human leukocyte antigen molecule (HLA) DRB1 and complement C4 alleles may underlie a genetic predisposition to neuroimmune dysfunction in autism (Ashwood et al. 2006; Ashwood and Van de Water, 2004; Sperner-Unterweger, 2006). The contribution of glial cell dysfunction to the pathogenesis of autism is another promising area of contemporary research (Aschner et al. 1999). On the whole, irregular immune activity during vulnerable periods of neurodevelopment may be the key to the neural dysfunction noted in autism (Ashwood et al. 2006; Cohly and Panja, 2005). Although these hypotheses carry potential credibility in the field of autism research, the fundamental link cementing the association between immunopathology and autism (and specifically its neuropsychiatric presentation) has yet to be ascertained. Furthermore, the general degree of uncertainty with regard to whether the immune insult is due to an autoimmune disturbance or an immune depressive event is an area which undoubtedly requires further investigation.

## Calcium Signalling

Calcium signalling may also contribute to autism via the important nature of activity-dependant calcium influx into neurons which subsequently regulates (via transcription) numerous cortical excitatory synapses (Shalizi et al. 2006). Altered calcium signalling could be a fundamental factor in the promotion of dysfunctional synaptogenesis and thus autism (Casanova, 2007; Pickett and London, 2005). It is possible that the observed defects in calcium signalling are also responsible for the unbalanced excitatory-inhibitory networks discussed earlier, rather than the GABA/glutamate receptor abnormalities.

## Mirror Neurone System Theory

The favourable mirror neurone system theory links neuropathology to autism on a more symptomatic level. The mirror neurons themselves are pre-motor and parietal cells of the cerebral cortex that fire action potentials not just when we are in action or motion, but when we observe others performing similar actions. These neurons essentially ‘mirror’ the behaviour of others and provide a suitable physiological mechanism for the great scope of social behaviours and skills that humans implement (i.e. imitation, modelling, empathy and language acquisition/evolution). Thus, mirror neurone dysfunction could be the central deficit observed in autism leading to the profound social and communication difficulties experienced (Iacoboni and Dapretto, 2006; Schmitz and Rezaie, 2008; Williams et al. 2001). Although the mirror neurone theory paints a promising view of a simple pathogenesis, the connection between the neural deficit and autism is likely to be speculative at best, as it is unlikely that such a small population of neurons can account for the great array of psychosocial and behavioural disturbances encountered in autistic children. This point is further enforced by the fact that other autistic behaviours (i.e. sensory hypersensitivity and excelling at visual tasks) cannot be explained by dysfunctional mirror neurones (Dinstein, 2008).

## Other Theories

Aside from the above theories there are several other lines of research into the pathogenesis of autism. One such theory involves decreased levels of apoptosis (programmed cell death) since subnormal levels of Bcl-2 and p53 protein have been documented in the cortices of autistic brains (Araghi-Niknam and Fatemi, 2003). Whether or not the subsequently inappropriate high level of neurons interferes in specific way with normal brain development is unknown to date, since currently there is a lack of research in the field of anti-apoptotic insults. However, it does seem probable that excess neurone numbers secondary to the disordered apoptosis, may promote defects in neural connectivity and communication (as detailed earlier). Neurotransmitter abnormalities involving higher than normal serotonin levels have also been implicated in the pathogenesis but with no certain link to autism on the symptomatic level. The cause of such elevated levels appears to be linked to variations in the genes (SLC6A4) coding for the serotonin transporter (Penn, 2006; Pino et al. 2004). Metabolic defects have also been associated with autism suggesting inborn errors of metabolism as a potential pathogenic factor (Manzi et al. 2008). Deficits in cell adhesion molecules, second messenger systems and secreted molecules have also all been implicated in autism’s rather complex pathophysiology (Persico and Bourgeron, 2006).

## Summary and Future Directions

It is clear from the above mentioned theories that there is not a direct pathway of mechanisms pointing towards a simple pathogenesis and an explanation of autism’s rather distinctive set of symptoms. It seems much more favourable to summarise autism’s pathogenesis as being multidimensional and uncertain but yet promising with regard to some of the aforementioned theories. These include: disturbed neural connectivity; defective neural migration; unbalanced excitatory-inhibitory networks; abnormal dendritic morphology; neuroimmune disturbances; calcium signalling and the mirror neurone system theory. Further research in the field of autism pathogenesis should focus on elucidating whether exactly the excitation abnormality is due to GABA receptor activity suppression or glutamate receptor activity elevation or defects in calcium signalling?; whether the neuroimmune pathogenic element is due to suppression of immune responses or more related to autoimmune insults?; why the mirror neurone theory cannot explain the great array of behavioural and psychosocial symptoms seen in autism?; what specific relevance disturbed neural connectivity has to autism, and whether there is a valid anti-apoptotic link? and finally how all the mentioned pathogenic factors interact together (if they do at all) to produce a deficit in the functional brain unit?

Overall it seems most likely that the anomaly encountered in neurodevelopment that leads to autism is not merely ‘localised’ to a single functional neural defect as many theories have suggested, but more distributed across numerous levels of study (genetic, immunopathogenic, neurofunctional etc.) becoming a ‘multi-domain’ disorder (Casanova, 2007; Muller, 2007; Persico and Bourgeron, 2006). Furthermore, it is worth mentioning that of all the theories of autism’s pathogenesis, the mirror neurone system theory at least provides us with an apparent positive connection at the symptomatic level. Future investigation into all the theories highlighted in this short review could facilitate the development of more efficacious treatments (pharmacological and non-pharmacological) for this complex developmental disorder.

## References

[b1-cpath-1-2008-099] Araghi-Niknam M, Fatemi SH (2003). Levels of Bcl-2 and P53 are altered in superior frontal and cerebellar cortices of autistic subjects. Cell Mol Neurobiol.

[b2-cpath-1-2008-099] Aschner M, Allen JW, Kimelberg HK (1999). Glial cells in neurotoxicity development. Annu Rev Pharmacol Toxicol.

[b3-cpath-1-2008-099] Ashwood P, van de Water J (2004). Is autism an autoimmune disease?. Autoimmun Rev.

[b4-cpath-1-2008-099] Ashwood P, Wills S, Van de Water J (2006). The immune response in autism: a new frontier for autism research. J Leukoc Biol.

[b5-cpath-1-2008-099] Boeckers TM, Bockmann J, Kreutz MR, Gundelfinger ED (2002). ProSAP/Shank proteins—a family of higher order organizing molecules of the postsynaptic density with an emerging role in human neurological disease. J Neurochem.

[b6-cpath-1-2008-099] Cannell JJ (2007). Autism and vitamin D. Med Hypotheses.

[b7-cpath-1-2008-099] Casanova MF (2007). The neuropathology of autism. Brain Pathol.

[b8-cpath-1-2008-099] Cohly HH, Panja A (2005). Immunological findings in autism. Int Rev Neurobiol.

[b9-cpath-1-2008-099] Courchesne E, Pierce K, Schumann CM (2007). Mapping early brain development in autism. Neuron.

[b10-cpath-1-2008-099] Davidson PW, Myers GJ, Weiss B (2004). Mercury exposure and child development outcomes. Pediatrics.

[b11-cpath-1-2008-099] Dinstein I, Thomas C, Behrmann M (2008). A mirror up to nature. Curr Biol.

[b12-cpath-1-2008-099] Dykens EM, Sutcliffe JS, Levitt P (2004). Autism and 15q11–q13 disorders: behavioral, genetic, and pathophysiological issues. Ment Retard Dev Disabil Res Rev.

[b13-cpath-1-2008-099] Fombonne E (2005). Epidemiology of autistic disorder and other pervasive developmental disorders. J Clin Psychiatry.

[b14-cpath-1-2008-099] Freitag CM (2007). The genetics of autistic disorders and its clinical relevance: a review of the literature. Mol Psychiatry.

[b15-cpath-1-2008-099] Lacoboni M, Dapretto M (2006). The mirror neuron system and the consequences of its dysfunction. Nat Rev Neurosci.

[b16-cpath-1-2008-099] Johnson TW (2006). Dietary considerations in autism: identifying a reasonable approach. Top Clin Nutr.

[b17-cpath-1-2008-099] Just MA, Cherkassky VL, Keller TA (2007). Functional and anatomical cortical underconnectivity in autism: evidence from an FMRI study of an executive function task and corpus callosum morphometry. Cereb Cortex.

[b18-cpath-1-2008-099] Kanner L (1943). Autistic disturbances of affective contact. Nervous Child.

[b19-cpath-1-2008-099] Kern JK, Jones AM (2006). Evidence of toxicity, oxidative stress, and neuronal insult in autism. J Toxicol Environ Health B Crit Rev.

[b20-cpath-1-2008-099] Kolevzon A, Gross R, Reichenberg A (2007). Prenatal and perinatal risk factors for autism. Arch Pediatr Adolesc Med.

[b21-cpath-1-2008-099] Magdaleno S, Keshvara L, Curran T (2002). Rescue of ataxia and preplate splitting by ectopic expression of Reelin in reeler mice. Neuron.

[b22-cpath-1-2008-099] Manzi B, Loizzo AL, Giana G, Curatolo P (2008). Autism and metabolic diseases. J Child Neurol.

[b23-cpath-1-2008-099] Miall L, Rudolf M, Levene M (2007). Paediatrics at a glance.

[b24-cpath-1-2008-099] Minshew NJ, Williams DL (2007). The new neurobiology of autism: cortex, connectivity, and neuronal organization. Arch Neurol.

[b25-cpath-1-2008-099] Muller RA (2007). The study of autism as a distributed disorder. Ment Retard Dev Disabil Res Rev.

[b26-cpath-1-2008-099] Penn HE (2006). Neurobiological correlates of autism: a review of recent research. Child Neuropsychol.

[b27-cpath-1-2008-099] Persico AM, Bourgeron T (2006). Searching for ways out of the autism maze: genetic, epigenetic and environmental clues. Trends Neurosci.

[b28-cpath-1-2008-099] Pickett J, London E (2005). The neuropathology of autism: a review. J Neuropathol Exp Neurol.

[b29-cpath-1-2008-099] Pino GD, Moessner R, Lesch KP (2004). Roles for serotonin in neurodevelopment more than just neural transmission. Curr Neuropharmacol.

[b30-cpath-1-2008-099] Schmitz C, Rezaie P (2008). The neuropathology of autism: where do we stand?. Neuropathol Appl Neurobiol.

[b31-cpath-1-2008-099] Schultz RT (2005). Developmental deficits in social perception in autism: the role of the amygdala and fusiform face area. Int J Dev Neurosci.

[b32-cpath-1-2008-099] Shalizi A, Gaudillière B, Yuan Z (2006). A calcium-regulated MEF2 sumoylation switch controls postsynaptic differentiation. Science.

[b33-cpath-1-2008-099] Sperner-Unterweger B, Winkler C, Fuchs D (2006). Immune. activation in autism. Pediatr. Neurol..

[b34-cpath-1-2008-099] Trottier G, Srivastava L, Walker CD (1999). Etiology of infantile autism: a review of recent advances in genetic and neurobiological research. J Psychiatry Neurosci.

[b35-cpath-1-2008-099] Wakefield AJ, Murch SH, Anthony A (1998). Ileal-lymphoid-nodular hyperplasia, non-specific colitis, and pervasive developmental disorder in children. Lancet.

[b36-cpath-1-2008-099] Williams JH, Whiten A, Suddendorf T (2001). Imitation, mirror neurons and autism. Neurosci Biobehav Rev.

[b37-cpath-1-2008-099] World Health Organization (2005). The International Statistical Classification of Diseases and Related Health Problems ICD-10. 10th Rev.

